# Chuzan Virus in Yaks, Qinghai-Tibetan Plateau, China

**DOI:** 10.3201/eid2412.171414

**Published:** 2018-12

**Authors:** Meng Wang, Yun Wang, Abdul Rasheed Baloch, Yangyang Pan, Lili Tian, Fang Xu, Shaobo Chen, Qiaoying Zeng

**Affiliations:** Gansu Agricultural University, Lanzhou, China (M. Wang, Y. Pan, F. Xu, Q. Zeng);; Anning Branch Lanzhou General Hospital, Lanzhou (Y. Wang);; University of South Bohemia in České Budějovice, Zátiší Vodňany, Czech Republic (A.R. Baloch);; China Animal Health and Epidemiology Center, Qingdao, China (L. Tian);; Veterinary Department of Gansu Province, Lanzhou (S. Chen)

**Keywords:** CHUV, Chuzan virus, yak, Qinghai-Tibetan Plateau, China, viruses, Orbivirus, Reoviridae

## Abstract

We detected Chuzan virus (CHUV) in domestic yaks from the Qinghai-Tibetan Plateau, western China, indicating CHUV probably has been transmitted to yaks in recent years. Awareness for CHUV surveillance and transmission and livestock health management in these special regions should be raised to avoid outbreaks and animal loss.

Chuzan virus (CHUV) belongs to the Palyam serogroup of genus *Orbivirus*, family *Reoviridae*. A CHUV outbreak, first reported in Japan in 1985, was the causative agent of disease that resulted in many reproductive disorders in cattle, including abortion, stillbirth, and congenital malformation (*1*) and in considerable economic loss in the cattle industry.

Like other orbiviruses, CHUV consists of 10 double-stranded RNA segments (Seg-1 to Seg-10), which encode 7 structural viral proteins (VP1–VP7) and 4 nonstructural proteins (NS1–NS4) (*2**–**4*). Seg-2 (VP2) and Seg-6 (VP5) of the Palyam serogroup show the highest levels of variation in genome sequence, which correlates with virus serotype specificity (*2*). These viruses usually are transmitted by arthropod vectors (*5**,**6*). Therefore, CHUV has been widespread in many countries of Asia, such as South Korea (*7*) and mainland China (*8**,**9*), which have reported CHUV infection in cattle. However, no information was available about CHUV in yaks (*Bos grunniens*) on China’s Qinghai-Tibetan Plateau.

Yaks are an important livestock in the Qinghai-Tibetan Plateau. They have been farmed with other livestock, such as Tibetan sheep and Tibetan pigs. The high prevalence of bluetongue virus (BTV) infection, also belonging to genus *Orbivirus*, has been reported in yaks and Tibetan sheep (*10*). A study in 2016 found an abortion rate in yaks of 21.39% in part of Qinghai Province, presumably because of the high prevalence of BTV and other related pathogens (*10*). All these data and CHUV infection in cattle in China motivated us to study whether CHUV infects yaks.

During August 2016–April 2017, we randomly collected 208 blood samples from apparently healthy domestic yaks, 71 yaks from Gansu Province (46 <1 year of age), 64 yaks from Qinghai Province (23 <1 year of age), and 73 yaks from Sichuan Province (29 <1 year of age). Soon after sampling, total RNAs were extracted and used as templates to amplify full-length cDNA by reverse transcription PCR (RT-PCR; SuperScript III Synthesis Kit, Invitrogen, Carlsbad, CA, USA). One pair of specific primers was designed based on VP2 genome sequence of CHUV (Technical Appendix Table 1) and used to detect CHUV in yaks. We also performed serologic assay by using the CHUV 2nd detection kit (iNtRON, IPC11028, Gyeonggi-do, South Korea), and the results of the assay were then authenticated by RT-PCR.

For phylogenetic and identity analysis of genome sequence of 10 segments from CHUV, we designed 10 pairs of primer based on known sequences deposited in GenBank (Technical Appendix Table 1) to obtain the open reading frame genome of these proteins. Phylogenetic and identity analyses were performed based on these genome sequences and the corresponding sequences available in GenBank.

Five (7%) of the 71 samples were positive for CHUV in Gansu Province, 4 of which were <1 year of age (Figure; Technical Appendix Table 2). CHUV infection in yaks was not found in Qinghai and Sichuan provinces. Relatively low prevalence of CHUV infection in yaks is consistent with the report of CHUV infection in cattle in South Korea (*7*), which was also significantly lower than BTV infection in yaks (17.34%) (*10*). We also obtained similar results in serologic investigation. We observed neither illness nor disease in these yaks; our results are consistent with CHUV infection in sentinel cattle (*8*) and thus suggest that subclinical infection of CHUV occurs in cattle. We used heparinized blood samples to inoculate baby hamster kidney 21 cells for 5 blind passages, as described previously (*6**,**8**,**9*). Infected cells that exhibited a wrinkled morphology and were detached from the bottom of culture flasks within 5 days (Technical Appendix Figure 1), and 19 segments of CHUV could also be detected by RT-PCR.

**Figure:**
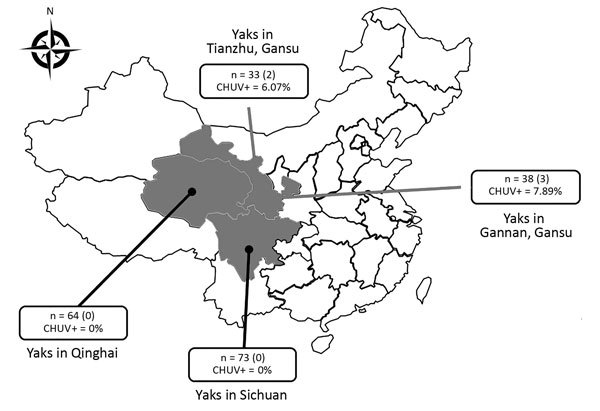
Number and species of yaks from provinces around the Qinghai Tibetan Plateau, China, 2016–2017. The 3 provinces where sampling was performed, yak species, and occurrence of CHUV are indicated. n values indicate the total number of samples in each province; numbers in parentheses indicate the numbers of positive samples in each province; CHUV+ percentages indicate the CHUV prevalence rate. CHUV, Chuzan virus.

Sequence analysis revealed 100% identity of genomes for Seg-1 to Seg-10 of 3 CHUV sequences in yaks (CHN-GS-70). Identity analysis showed that genome sequences for Seg-1 to Seg-10 of CHUV shared >98.38% nt identities and >98.09% aa identities with CHUV strain KT887181/GX871/China in previous studies (*8**,**9*) (online Technical Appendix Table 3). We constructed 2 phylogenetic trees based on VP2 and VP5 genome sequences of CHUV and other members in genus *Orbivirus* (Technical Appendix Figures 2, 3). All strains from our study were grouped in a new separate cluster and shared an ancestor with the strains KT002589/SZ187/China and KT887181/GX871/China. Furthermore, CHN-GS-26 and CHN-GS-70 were located in the same separate subcluster (Technical Appendix Figures 2, 3), which demonstrated a complicated and transregional transmission cycle for CHUV in China.

The yaks that were positive for CHUV were located in 2 cities of Gansu Province ≈600 km apart, which indicates that transmission of CHUV has spread rapidly around the Qinghai-Tibetan Plateau. Further studies are needed to determine the epidemiology and evolution of CHUV in livestock with concomitant virus isolation and phylogenetic analysis. The awareness of livestock health management in these special regions should also be raised.

Technical Appendix Additional methods and results for study of Chuzan virus in yaks, Qinghai-Tibetan Plateau, China, 2016–2017.
