# Supplementary material for: Chuzan Virus in Yaks, Qinghai-Tibetan Plateau, China
Source: Emerg Infect Dis. 2018 Dec;24(12):2371–3. doi: 10.3201/eid2412.171414 (PMC6256394; doi:10.3201/eid2412.171414)
Supplement: Technical Appendix — Additional methods and results for study of Chuzan virus in yaks, Qinghai-Tibetan Plateau, China, 2016–2017. [file 17-1414-Techapp-s1.pdf]

Article DOI: https://doi.org/10.3201/eid2412.171414

Chuzan Virus in Yaks, Qinghai-Tibetan Plateau, China

Technical Appendix

Technical Appendix Table 1. Primers used for CHUV detection and full-length genome amplification of segments 1–10*

Primers	Sequences	Length, bp	Function†	Reference‡
CHUV-D-F	5′-GTGTTCAGACTGATAACATCATCG-3′	364	CHUV detection	KT887181,
CHUV-D-R	5′-GCTGGACTGTTGATTATCCCTC-3′			AB014725
CHUV-Seg-1-F	5′-GGTCAATCATGCAAGACGCATC-3′	3903	VP1 amplification	KT887180
CHUV-Seg-1-R	5′-GGTGACGCTAAACAAATTGATTCTG-3′			
CHUV-Seg-2-F	5′-TTCCGCAATGGATGAATTTTCG-3′	3034	VP2 amplification	KT002589
CHUV-Seg-2-R	5′-GCAACACGTAGTTGTACACTACG-3′			
CHUV-Seg-3-F	5′-TGTAGGATGGATGCTCAACGTA-3′	2736	VP3 amplification	KT887182
CHUV-Seg-3-R	5′-ACAGGTTGGTCAGTCCTATACAG-3′			
CHUV-Seg-4-F	5′-CATGGAGCCTTGTGCAGTGTTAC-3′	1938	VP4 amplification	KT887183
CHUV-Seg-4-R	5′-CCTTCGAGTTTCACCTACTCTT-3′			
CHUV-Seg-5-F	5′-CGTTTCAGAATGGAACGATTCC-3′	1719	NS1 amplification	KT887184
CHUV-Seg-5-R	5′-CACCGAACGATCCTAACCTAAAC-3′			
CHUV-Seg-6-F	5′-GAACACGATGGGTCGGTTCGTGA-3′	1593	VP5 amplification	KT002593
CHUV-Seg-6-R	5′-GCTGATTTGCTCCTGACCGACT –3′			
CHUV-Seg-7-F	5′-TCTCCTCGAGATGGATGCGATTG-3′	1102	VP5 amplification	KT887186
CHUV-Seg-7-R	5′-ATCTAGTGTGACTGATGCATTGTG-3′			
CHUV-Seg-8-F	5′-CCTTGACATCATGGGTGACAG-3′	1024	NS2 amplification	KT887187
CHUV-Seg-8-R	5′-CCTATCAGTCAACTAGTGGACAG-3′			
CHUV-Seg-9-F	5′-GTTGTGGTTGATGACGACGTCTC-3′	839	VP6 amplification	KT887188
CHUV-Seg-9-R	5′-CGTGCCAATCCTACATATGATC-3′			
CHUV-Seg-10-F	5′-GAAATGTTGGCGCGGTCGCTA-3′	662	NS3 amplification	KT887189
CHUV-Seg-10-R	5′-CGTTCGGAATCCAATACTCG-3′			

*CHUV, Chuzan virus; D detection; NS, nonstructural protein; F, forward; R, reverse; seg, segment; VP, viral protein.

†The aim of the primers used in this study.

‡The reference used for specific primers designation.

Technical Appendix Table 2. Results of Chuzan virus detection in yaks by reverse transcription PCR for 3 provinces surrounding Qinghai Tibetan Plateau, China, 2016–2017

		No*			
Province	Age, mo.	2016	2017	No. positive (%)†	Total positive (%)‡
Gansu	≤1	35	11	4/46 (8.7)	5/71 (7.04)
	>1	13	12	1/25 (4.0)	
Qinghai	≤1	8	15	0/23 (0)	0/64 (0)
	>1	27	14	0/41 (0)	
Sichuan	≤1	14	15	0/29 (0)	0/73 (0)
	>1	21	23	0/44 (0)	

*The number of yak samples collected from each province with 2 age groups at various times (2016–2017) in the study.

†Number and percentage of positive samples in each age group of different provinces.

‡Number and percentage of positive samples in all age groups of different provinces.

Technical Appendix Table 3. Nucleotide and amino acid identities of segments 1–10 of CHUV discovered in this study with the strain GX871/China deposited in GenBank*

CHUV segment	CHUV discovered in this study	CHUV/GX871/China	Nucleotide/amino acid, %
Seg-1	MH090056/CHN-GS-16	KT887180/GX871/China	99.59/99.07
	MH090057/CHN-GS-26		99.48/98.83
	MH090058CHN-GS-70		99.43/98.88
Seg-2	MF642326/CHN-GS-16	KT887181/GX871/China	99.03/98.76
	MF642327/CHN-GS-26		98.83/98.29
	MF642328/CHN-GS-70		98.69/98.09
Seg-3	MH090059/CHN-GS-16	KT887182/GX871/China	99.33/98.65
	MH090060/CHN-GS-26		99.41/98.65
	MH090061/CHN-GS-70		99.56/99.02
Seg-4	MH090062/CHN-GS-16	KT887183/GX871/China	99.43/98.90
	MH090063/CHN-GS-26		99.27/98.43
	MH090064/CHN-GS-70		99.32/98.43
Seg-5	MH090065/CHN-GS-16	KT887184/GX871/China	99.96/98.90
	MH090066/CHN-GS-26		99.51/98.43
	MH090067/CHN-GS-70		99.51/98.43
Seg-6	MF642329/CHN-GS-16	KT887185/GX871/China	98.91/99.08
	MF642330/CHN-GS-26		98.65/98.71
	MF642331/CHN-GS-70		98.38/98.71
Seg-7	MH090068/CHN-GS-16	KT887186/GX871/China	99.71/99.71
	MH090069/CHN-GS-26		99.81/100.00
	MH090070/CHN-GS-70		99.81/99.71

CHUV segment	CHUV discovered in this study	CHUV/GX871/China	Nucleotide/amino acid, %
Seg-8	MH090071/CHN-GS-16	KT887187/GX871/China	99.50/99.40
	MH090072/CHN-GS-26		99.70/99.10
	MH090073/CHN-GS-70		99.70/99.10
Seg-9	MH090074/CHN-GS-16	KT887188/GX871/China	99.88/99.63
	MH090075/CHN-GS-26		99.51/98.89
	MH090076/CHN-GS-70		99.51/98.89
Seg-10	MH090077/CHN-GS-16	KT887189/GX871/China	99.68/99.52
	MH090078/CHN-GS-26		99.68/100.00
	MH090079/CHN-GS-70		99.53/99.05

*The newly identified CHUVs sequence surrounding Qinghai-Tibet Plateau of China, and have been deposited in GenBank. CHUV, Chuzan virus; seg, segment.

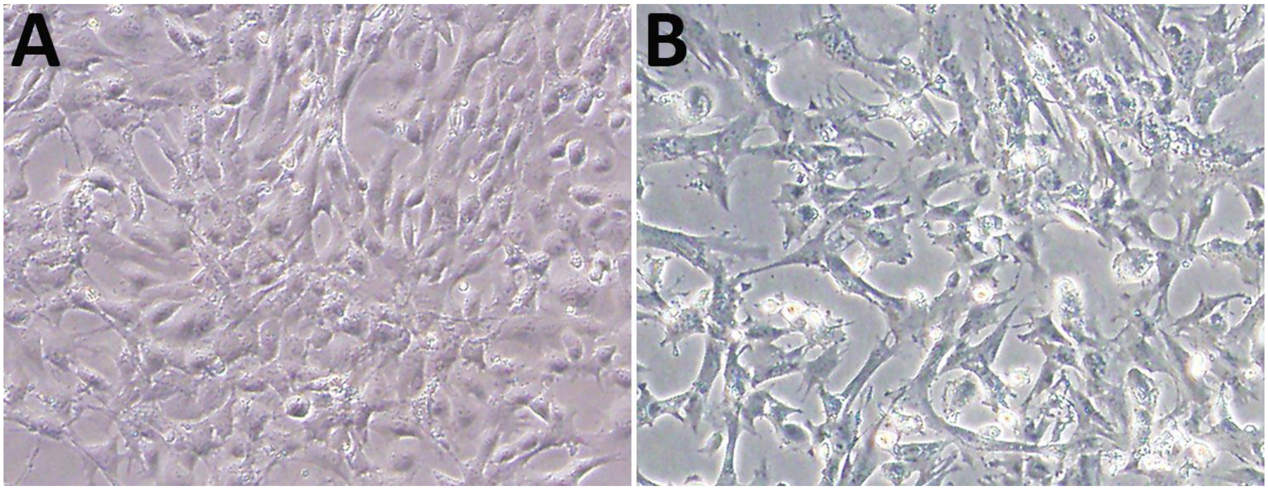

Technical Appendix Figure 1. Cytopathic effects of baby hamster kidney (BHK-21) cells that were inoculated with Chuzan virus (CHUV). (A) BHK-21 cells at fifth passage without CHUV infection. Original magnification ×200. (B) BHK-21 cells at fifth passage infected with CHUV at first passage. The infected cells that exhibited a wrinkled morphology and were detached from the bottom of culture flasks. Original magnification ×200.

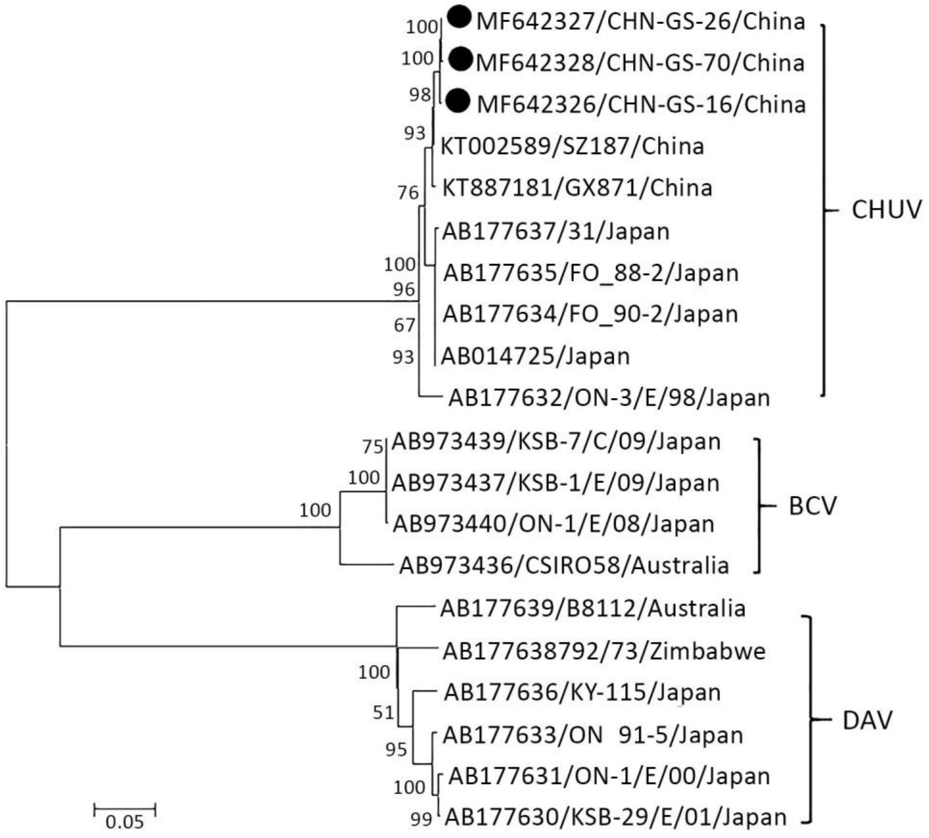

Technical Appendix Figure 2 Phylogenetic analysis of CHUV based on viral protein 2 genome sequences of CHUV and other members in Palyam serogroup of genus *Orbivirus* available in August 2017. The phylogenetic tree was constructed by using the neighbor-joining method with MEGA 7.01 software (http://www.megasoftware.net). Bootstrap values were calculated with 1,000 replicates. The number on each branch indicates bootstrap values. Black circles indicate the newly identified CHUVs sequence surrounding Qinghai-Tibet Plateau of China, and have been deposited in GenBank (GenBank accession no. MF642326/CHN-GS-16, MF642327/CHN-GS-26 and MF642328/CHN-GS-70). The reference sequences obtained from GenBank are indicated by GenBank accession numbers, strain abbreviations and countries. Scale bar indicates nucleotide substitutions per site. BCV, Bunyip Creek virus; CHUV, Chuzan virus; DAV, D’Aguilar virus.

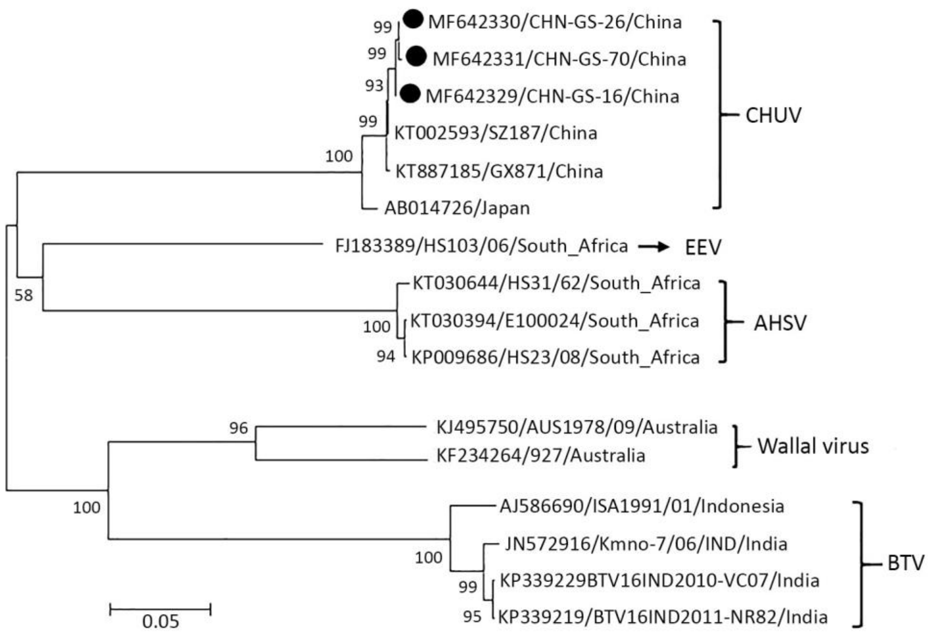

Technical Appendix Figure 3 Phylogenetic analysis of CHUV based on viral protein 5 genome sequences of CHUV and other members in genus *Orbivirus* available in August 2017. The phylogenetic tree was constructed by using the neighbor-joining method with MEGA 7.01 software (http://www.megasoftware.net). Bootstrap values were calculated with 1,000 replicates. The number on each branch indicates bootstrap values. Black circles indicate the newly identified CHUV sequence surrounding Qinghai-Tibet Plateau of China, which have been deposited in GenBank (GenBank accession no. MF642329/CHN-GS-16, MF642330/CHN-GS-26 and MF642331/CHN-GS-70). The reference sequences obtained from GenBank are indicated by GenBank accession numbers, strain abbreviations and countries. Scale bar indicates nucleotide substitutions per site. AHSV, African horse sickness virus; BTV, bluetongue virus; CHUV, Chuzan virus.
